# Finding the right doctoral thesis – an innovative research fair for medical students

**DOI:** 10.3205/zma000971

**Published:** 2015-08-17

**Authors:** Julius Steffen, Markus Grabbert, Tanja Pander, Maximilian Gradel, Lisa-Maria Köhler, Martin R. Fischer, Philip von der Borch, Konstantinos Dimitriadis

**Affiliations:** 1 Klinikum der Universität München, Institut für Didaktik und Ausbildungsforschung in der Medizin, München, Deutschland; 2 Klinikum der Universität München, Medizinische Klinik und Poliklinik I, München, Deutschland; 3 Klinikum der Universität München, Urologische Klinik und Poliklinik, München, Deutschland; 4 Klinikum der Universität München, Medizinische Klinik und Poliklinik IV, München, Deutschland; 5 Klinikum der Universität München, Neurologische Klinik und Poliklinik, München, Deutschland

**Keywords:** student research, doctoral thesis, research fair, CanMEDS

## Abstract

**Introduction: **The importance of research, as promoted by the *CanMEDS* framework, is widely acknowledged. Many medical students in Germany work on a research project as part of their doctoral thesis whilst still going to medical school. However, a significant amount of projects are abandoned unfinished, which leads to substantial wastage of resources. One reason for this is an information deficit concerning undergraduate research projects.

**Project description: **To counteract this, we introduced an annual event at LMU Munich called *DoktaMed* with more than 600 visitors each year. It combines medical convention and research fair including keynote lectures, workshops and poster sessions as well as an exhibition of research groups and institutes. *DoktaMed* is a peer-to-peer event organized by a team of 40 students.

**Results:** A needs analysis before its implementation underlined the information deficit as a possible cause for the high rate of abandoned projects. In the annual evaluation, visitors of *DoktaMed* rate the event with an average grade of 2.1 on a six-level Likert scale (n=558, SD=1.06, with "1=very good", "6=poor"). They stated to now feel better informed about the topic and regarded visiting *DoktaMed* as a worthwhile investment of time.

**Discussion:** Students are generally satisfied with the event and feel better informed after visiting *DoktaMed*. However, many students never visit DoktaMed for various reasons. A possible improvement would be to present a greater number of clinical studies in addition to the laboratory work that *DoktaMed* focuses on now.

**Conclusion: **Evaluation after six years of *DoktaMed* is very promising. Visitors seem to be better informed. Nevertheless there is space for improvement in order to get more students and more faculty members involved. More studies are needed to assess long-term effects.

## Zusammenfassung

**Einleitung: **Die Forschung in der Medizin, wie sie auch im Rahmen des *CanMEDS* Modell beschrieben wird, genießt einen hohen Stellenwert. Viele Medizinstudierende in Deutschland beginnen schon während des Studiums mit der Arbeit an einem Promotionsprojekt. Eine große Anzahl dieser Projekte wird allerdings oft unvollendet abgebrochen, wodurch unnötig Ressourcen verschwendet werden. Ein Hauptgrund hierfür ist ein Mangel an Informationen über studentische Forschungsprojekte.

**Projektbeschreibung: **Um diesem Mangel entgegenzuwirken, haben wir an der LMU München die jährlich stattfindende Veranstaltung *DoktaMed* ins Leben gerufen. Die Kombination von medizinischem Fachkongress und Forschungsmesse mit Vorträgen, Poster-Präsentationen und Workshops sowie einer großen Ausstellung der verschiedenen Arbeitsgruppen und Institute der LMU zieht jedes Jahr mehr als 500 Besucher an. Organisiert wird die *DoktaMed* als Peer-to-Peer-Veranstaltung von einem Team aus etwa 40 Studierenden.

**Ergebnisse: **Eine Bedarfsanalyse vor der Einführung der *DoktaMed* identifizierte ein Informationsdefizit als eine wichtige Ursache für die hohe Rate abgebrochener Doktorarbeiten. In den jährlichen Evaluationen durch die Besucher der *DoktaMed* wird die Veranstaltung durchschnittlich mit der Note 2,1 auf einer sechsstufigen Likert-Skala bewertet (n=558, SD=1,06, 1=sehr gut, 6=sehr schlecht). Die Studierenden empfinden ihren Besuch auf der *DoktaMed* als sinnvoll investierte Zeit und fühlen sich anschließend besser zum Thema Doktorarbeit informiert.

**Diskussion:** Insgesamt sind die Studierenden mit der Veranstaltung zufrieden und fühlen sich besser auf die Doktorarbeitssuche vorbereitet. Dennoch besuchen viele Studierende die Messe aus unterschiedlichen Gründen nicht. Eine Möglichkeit zur weiteren Verbesserung besteht darin, zusätzlich zu den experimentellen Arbeiten, auf die sich die *DoktaMed* momentan konzentriert, ein breiteres Angebot an klinischen Projekten zu präsentieren..

**Schlussfolgerung: **Die Evaluation nach fünf Jahren *DoktaMed* sieht vielversprechend aus. Die Besucher scheinen tatsächlich besser zum Thema Doktorarbeit informiert zu sein. Trotzdem existieren Möglichkeiten zur weiteren Verbesserung, um die *DoktaMed* für Studierende und Fakultätsmitglieder attraktiver zu gestalten. Zur Beurteilung von Langzeiteffekten sind weitere Untersuchungen notwendig.

## Introduction

In contrast to many other countries, German universities award the academic title “doctor of medicine” (Dr. med.) as a research doctorate. Conducting a research project and submitting a thesis is mandatory [1]. Almost all medical students start such a research project before graduation but only three quarters of German physicians have actually finished their doctoral thesis 10 years after graduation [2].

The physician’s role as a scholar as represented in the *CanMEDS*-framework [3] is widely acknowledged among doctors of different specialties. Boyer identifies innovation as a key scholarly activity that every medical student should experience through a research project [4]. Nevertheless, teaching and learning research skills are not yet regular parts of medical curricula in many countries [5].

The scientific importance and methodical quality of German doctoral theses have been a matter of controversial debate for many years [6], [7]. About 15% of doctoral students abandon their research projects even before graduation [8], [9]. Lack of support, non-availability of the supervisor, conceptual flaws of the project, no recognizable progress, an unacceptable workload or a lack of time are major reasons for unsuccessful doctoral projects [9], [10]. More than half of the students working on a thesis are not familiar with the doctoral regulations of their universities. Moreover, 76% of students felt insufficiently prepared for research by their medical schools [11]. 

Hypothesizing a lack of information before choosing a research project as the major reason for an unsuccessful or burdensome experience with medical doctoral theses projects, we conducted a quantitative and qualitative needs analysis at LMU Munich to investigate this issue. Then, based on the results, we launched an annual research fair at our faculty, called *DoktaMed*. This article aims to present this innovative fair to a wider audience and to discuss first evaluation results six years after its introduction.

## Project Description

*DoktaMed *is an annual event limited to LMU Munich combining a medical convention and research fair including keynote lectures, oral presentations of research topics conducted by selected faculty members and students, poster presentations and an exhibition of research groups. The main goals of *DoktaMed* are to inform students about the structure, opportunities and pitfalls of doctoral theses in general, enabling them to make informed decisions when opting to get involved in research projects. Additionally, *DoktaMed* is a great occasion to practice presentation of scientific results. As a side goal, we sought to increase the exchange of ideas between research groups. 

### What is DoktaMed composed of?

The research fair component of *DoktaMed* comprises about half of the 60 institutes of LMU medical faculty, each represented in their own booths. Professors, junior faculty scientists as well as fellow doctoral students are available here to answer visitors’ questions thereby not intending to make specific offerings to prospect doctoral students but to rather provide general information and their personal view on student research. Guided tours, so called *DoktaWalks*, around the booths help students overcome potential timidity addressing senior faculty members. In parallel to the fair, selected faculty members and doctoral candidates hold keynote lectures. 

The other major component of *DoktaMed *is its resemblance of a scientific conference. Each year, students currently involved in their medical doctoral theses are encouraged to submit an abstract that is being reviewed by a jury comprised of senior faculty as well as fellow students. This way, they are given the opportunity to practice academic writing as well as to present their data in oral presentations or poster sessions. Thus, interested students have the chance to get first-hand information about research groups or the processes involved in pursuing the doctoral title from peers. 

#### Set up

*DoktaMed* used to take place on two consecutive weekdays for the first two years (2009 & 2010) until, encouraged by evaluation results, it was moved and compressed to a Saturday. It is complemented by workshops concerning topics such as academic writing, translational medicine, software commonly used in research and presentation skills. The workshops used to be scheduled on the day of the fair but are now taking place in the days and weeks before and after, allowing them to actually prepare students for their presentations held at *DoktaMed* and preventing scheduling conflicts.

#### Costs and organization

The total cost of booth rental, catering, printing of booklets, posters and flyers comprises around 10,000€ annually. While the medical faculty kindly provided financial means in the first years, donations account for most of the funds now. 

*DoktaMed* is a peer-to-peer event and is organized by a team of 40 volunteering students. Students are recruited via an email invitation about six months prior to the event. The mentoring office coordinates the team during the initial planning phase with formal support from the deans of research and studies. The organization team is organized in departments and subdivisions, such as logistics, marketing, finance, IT, abstract management, research group management, and workshops. Despite the increasing use of written documentation to thwart this issue, some procedural knowledge is frequently lost year over year and leads to obstacles that might have been overcome in years past. This is something we plan to improve, however the constant fluctuation in the student body makes this task exceedingly difficult.

Visitors evaluated each DoktaMed via an online survey sent to all medical students right after the fair as well as a paper version distributed at the fair itself.

The program of the fair, a list of participating working groups and a lot of other information regarding doctoral theses can be found on the *DoktaMed* homepage (http://doktamed.de/). 

## Results

### Needs assessment

Before the implementation of *DoktaMed,* we performed a qualitative and quantitative needs analysis. In 2008, we conducted four focus group analyses with 7 to 8 participants each comparing students who had not yet started and students that were close to completion of their doctoral theses. All participants emphasized the importance of research for the medical profession on the one hand. One student said “a good doctor has to be able to correctly interpret newly published data”. On the other hand participants claimed to have difficulties in selecting a suitable research project for themselves because “there is no official information” as another participant put it. Based on these results, we developed an online questionnaire addressed to all 4,000 students enrolled at our medical faculty at LMU. 

The online survey underlined that there is clear information deficit. Students stated that they did not “feel well informed about medical theses by our faculty” with a mean grade of 4.34 (n=388, SD=1.30) on a six-level Likert scale (with 1=strongly agree and 6=strongly disagree) and hold this responsible for difficulties in selecting a suitable research project. As a possible solution, they suggested an informative event. 

#### Evaluation after 5 years

Since its introduction in 2009, more than 500 students visited *DoktaMed* each year. According to evaluation data, about two thirds of the visitors (n=298, 61.0%) have not yet started a doctoral thesis and are, on average, in their fourth semester (semester=4.22, SD=1.73). Visitors who are already working on a project (n=190, 39.0%) are usually in their ninth semester (semester=9.29, SD=2.16).

Evaluation showed that visitors generally rated *DoktaMed* with an average grade of 2.11 on a six-level Likert scale (n=558, SD=1.06, ranging from “1=very good” to “6=poor”). When asked for the most helpful component, about half of the visitors (n=298, 46.6%) named the booths as most beneficial, followed by the guided tours around the booths (n=147, 23.0%). After having been to *DoktaMed*, students state they are “better informed about the topic doctoral thesis” (n=513, mean=2.31, SD=1.38, ranging from “1=very good” to “6=poor”). In addition they “have a better understanding of the different kinds of doctoral projects” (n=370, mean=2.00, SD=1.08) and “know better, what kind of doctoral thesis they would like to do” (n=364, mean=2.23, SD=1.28). Overall, students thought that visiting *DoktaMed* was a worthwhile investment of time (n=528, mean=2.30, SD=1.41).

Similarly, professors and other faculty scientists attending the booths provided almost exclusively positive feedback. 

Nevertheless, about half the student body never visited DoktaMed (n=635, 51.2%). Among the reasons for not attending, “extracurricular events” (28.2%) and “studying for exams” (26.8%) account for the most. About a quarter also stated they were “already working on a doctoral thesis”.

## Discussion

Survey and evaluation data indicate students’ overall satisfaction with this event. Every year, students feel better informed by the faculty concerning doctoral theses after having visited *DoktaMed.* The rather informative components of the research fair, the booths of the different departments, divisions and institutes and the guided tours, were most useful to students according to the evaluation. Students estimated themselves to be better informed about doctoral theses in general and reported to have a better understanding of the different kinds of medical research. Specifically, after visiting *DoktaMed*, students assert they know better what kind of project they would like to do. This leads the authors to believe that *DoktaMed* actually helps students make better decisions about their thesis work, although further confirmation is desirable.

Nevertheless, only half the student body has visited *DoktaMed*. In the future, the date on which the event takes place should be planned more carefully to avoid an overlap with the students’ main study periods. Furthermore, we intend to attract more medical students already working on a project by putting more emphasis on information on career planning. 

Additionally, most projects presented at *DoktaMed* include laboratory work. Clinical studies, especially retrospective ones, are underrepresented. Projects of that type are regarded to be of lower quality, which we assume is the reason why doctoral students might feel embarrassed to be presenting their work at the fair. 

Unfortunately, with roughly 30 participating working groups each year, only half of the institutes of the medical faculty are represented at *DoktaMed*. Possible explanations include working groups having no capacity for further doctoral candidates, or faculty being unwilling to work unpaid overtime since *DoktaMed* usually takes place on weekends. Promoting more interaction between senior faculty and researchers from different groups could increase their interest in reserving a booth.

In addition to this, some faculty have a critical view on the event as working groups are often desiring more financial and idealistic support from the faculty and thus do not see a reason for supporting the faculty themselves. Anecdotally, some (predominantly older) faculty think along the lines of Darwin, saying students should not receive too much help.

Having noticed that students seek information all year round, we constructed an online database on our *DoktaMed* homepage where project leaders can upload thesis offerings and students can search, rank or filter them using different selection criteria (http://doktamed.de/doktaboerse). 

With a decline from 102 initially to only 40 abstracts submitted last year, increasing the number of students who take the opportunity to present their own research remains challenging. 

*DoktaMed* has the potential to counteract the information deficit concerning medical doctoral theses enabling the students to make better decisions when choosing a project. Thus, resources can be saved and the quality of research projects can be enhanced. Medical faculties should support initiatives like *DoktaMed* and open up a debate on how to improve medical doctoral theses and student research. At LMU, DoktaMed should be integrated into the medical curriculum to strengthen the physician’s role as a scholar as described in the CanMEDS framework. First steps to do so have already been undertaken.

## Conclusion

Based on our needs analysis and the evaluation data from six years of *DoktaMed*, we conclude that a lack of information is an essential issue hampering successful completion of medical doctoral theses in Germany. The implementation of a large-scale informative event like *DoktaMed*, while not entirely sufficient, seems to be a step in the right direction. So far, we cannot assess the effect *DoktaMed* has on quality and significance of doctoral theses. Although many German universities are currently assessing student research at their faculties it is hard to gather long-term information on the number of abandoned doctoral projects and to quantify the money and material wasted [12].

The format of a research fair could well be translated to other universities and is not at all limited to medical faculties but is applicable to other sciences and humanities as well. 

## Acknowledgements

The authors would like to thank all students involved in organizing *DoktaMed* every year. Special thanks go to Bernadette Bohn, Benedikt Blumberg and Florian Gothe. Thanks also go to the medical faculty of Ludwig-Maximilians-Universität, Munich, for their strong financial support.

## Competing interests

The authors declare that they have no competing interests.
